# Weekly HBsAg Decline Dynamics During Combination Therapy with Interferon and Nucleos(t)ide Analogs: A Retrospective Real-World Study

**DOI:** 10.3390/v18091006

**Published:** 2026-09-12

**Authors:** Ziyuan Yi, Zhuoran Huang, Caixia Duan, Xinru Hu, Dongyang Chen, Jie Zhang, Kuancheng Liu, Xiangjun Du, Caijun Sun, Guangyu Huang

**Affiliations:** 1 School of Public Health (Shenzhen), Sun Yat-Sen University, Shenzhen 518107, China; 2 Department of Infectious Diseases, The Seventh Affiliated Hospital, Sun Yat-Sen University, Shenzhen 518107, China; 3 Key Laboratory of Tropical Disease Control (Sun Yat-Sen University), Ministry of Education, Guangzhou 514400, China; 4 Shenzhen Key Laboratory of Pathogenic Microbes and Biosafety, Shenzhen Campus of Sun Yat-Sen University, Shenzhen 518107, China; 5 Department of Infectious Diseases, The Fourth Affiliated Hospital Zhejiang University School of Medicine, Yiwu 322000, China

**Keywords:** hepatitis B surface antigens, hepatitis, chronic, kinetics

## Abstract

Functional cure, defined as sustained hepatitis B surface antigen (HBsAg) loss, remains a major goal in chronic hepatitis B (CHB) management. HBsAg kinetics during interferon (IFN)-based therapy are associated with treatment response and prognosis, but real-world data on weekly HBsAg dynamics remain limited. This retrospective real-world study enrolled 533 CHB patients treated with IFN monotherapy or IFN plus tenofovir disoproxil fumarate (TDF), tenofovir alafenamide (TAF), or entecavir (ETV). Weekly HBsAg levels up to week 24 were analyzed using propensity score matching (PSM), subgroup analyses, and generalized estimating equations (GEEs). HBsAg kinetics showed marked heterogeneity, with some patients exhibiting transient early increases. Lower baseline HBsAg levels and rapid early decline were strongly associated with HBsAg loss. After PSM, IFN monotherapy and combination therapy showed comparable HBsAg decline trajectories. The three NA regimens demonstrated similar overall kinetics, although subgroup differences were observed by sex, immune activity, HBV DNA status, and prior treatment. TDF showed a faster decline in females, whereas ETV declined faster in males and immune-active patients. In conclusion, HBsAg kinetics during IFN-based therapy are highly heterogeneous and mainly influenced by baseline HBsAg levels, early on-treatment decline, and host factors. Weekly HBsAg monitoring may help predict treatment response and guide individualized CHB management.

## 1. Introduction

Hepatitis B virus (HBV) infection remains a major global public health challenge, particularly in developing countries such as China. Chronic hepatitis B (CHB) is a leading risk factor for liver cirrhosis and hepatocellular carcinoma (HCC). According to the World Health Organization (WHO), approximately 254 million individuals were living with chronic HBV infection in 2022, with 1.2 million new infections and 1.1 million deaths annually attributed predominantly to end-stage liver disease [1]. Thus, the primary objective of CHB treatment is to suppress viral replication and reduce the risk of cirrhosis and HCC. However, current antiviral therapies rarely achieve functional cure, which is characterized by sustained HBsAg loss and durable immune control after treatment cessation. Recent advances have shifted the therapeutic goal of CHB management from long-term viral suppression toward achieving functional cure [2].

Current antiviral therapies for CHB mainly consist of two drug classes: immunomodulators, such as pegylated interferon-alpha (Peg-IFN-α), and direct-acting antiviral agents, such as nucleos(t)ide analogs (NAs). However, due to the persistence of covalently closed circular DNA (cccDNA) in the nuclei of infected hepatocytes and the absence of agents that directly target cccDNA, complete viral eradication remains unattainable. This biological constraint has necessitated a shift in therapeutic goals from a sterilizing cure to a functional cure [3,4]. A key clinical efficacy indicator for assessing functional cure is the hepatitis B surface antigen (HBsAg) level, generally evaluated relative to baseline measurements. In clinical practice, combination therapy with Peg-IFN and NAs has increasingly been explored as a strategy to enhance HBsAg decline and increase the probability of functional cure, particularly among selected patients receiving long-term NA suppression [5]. However, previous comparisons of combination regimens have predominantly focused on post-treatment outcomes, with limited emphasis on HBsAg dynamics during therapy [6,7,8].

Extensive research has demonstrated a strong correlation between HBsAg levels and therapeutic efficacy. Early HBsAg decline during Peg-IFN therapy has been recognized as an important predictor of treatment response and functional cure. Recent meta-analytic evidence further demonstrated that HBsAg decline at week 24 provides strong predictive value for functional cure, supporting the clinical utility of longitudinal HBsAg monitoring [9]. For example, one retrospective study revealed that CHB patients with baseline HBsAg levels ≤ 25,000 IU/mL had significantly higher rates of hepatitis B e antigen (HBeAg) seroconversion six months after treatment discontinuation compared with those with higher baseline levels (35% vs. 16.3%, *p* < 0.0001) [10]. Another cohort study involving CHB patients with ultra-low baseline HBsAg levels who received short-term Peg-IFN-α-2b therapy identified baseline HBsAg as an independent predictor of on-treatment decline (OR = 0.984, *p* = 0.001) [11]. Furthermore, a recent study used extracellular viral markers, including HBsAg, to predict intracellular cccDNA kinetics [12], while another employed longitudinal discriminant analysis incorporating serial HBsAg quantification and clinical data to estimate the kinetics of HBsAg loss [13].

Although previous treatment guidelines did not typically consider patients in the inactive phase as standard candidates for antiviral therapy, long-term clinical practice and research have demonstrated that Peg-IFN-α-based regimens can achieve a relatively high HBsAg clearance rate (exceeding 40%) in this population [14,15,16]. The efficacy is profoundly influenced by the baseline HBsAg level, with clearance rates exceeding 80% or even reaching above 90% observed in patients with baseline HBsAg levels < 100 IU/mL, particularly those <10 IU/mL [15,16,17]. Extending the treatment duration can further improve the clinical cure rate [14], while dynamic changes in indicators such as baseline and on-treatment HBsAg levels serve as effective predictors of clinical cure [18]. Therefore, precise and timely monitoring of on-treatment HBsAg dynamics is essential for guiding therapeutic decisions.

In this study, we conducted a comprehensive analysis of the weekly decline rates of HBsAg across diverse treatment regimens using real-world clinical data. Our findings aim to provide evidence to support more accurate and individualized treatment strategies for patients with CHB.

## 2. Materials and Methods

### 2.1. Study Population

This study was approved by the Ethics Committee of the Seventh Affiliated Hospital of Sun Yat-sen University (Approval No. KY-2025-441-01). A retrospective pragmatic clinical study was conducted based on the institutional case database. From 30 May 2018 to 31 July 2024, a total of 1129 patients with CHB received interferon-based therapy. According to current guidelines [11], all patients met the indications for interferon treatment and voluntarily chose either interferon monotherapy or combination therapy with NAs. Pegylated interferon was administered in accordance with the prescribing information for Pegasys (Roche, Basel, Switzerland) or Pegberon (Xiamen Amoytop Biotech Co., Ltd., Xiamen, China). After screening according to predefined inclusion and exclusion criteria, a total of 533 eligible cases were ultimately included in the analysis.

Inclusion criteria: (1) age 18–65 years; (2) duration of HBV infection ≥ 6 months; and (3) achievement of HBsAg clearance at least once or completion of antiviral treatment for ≥24 weeks without regimen change. The ≥24-week requirement was intended to ensure a sufficient observation period for evaluating longitudinal HBsAg dynamics among patients who did not clear HBsAg, whereas patients with early clearance were kept because their endpoint event had already occurred, allowing us to include their complete trajectory data.

Exclusion criteria: (1) allergy to interferon; (2) decompensated cirrhosis; (3) white blood cell count < 3.5 × 10^9^/L and/or platelet count < 80 × 10^9^/L; (4) severe cardiovascular, renal, pulmonary, neurological, or ophthalmic diseases; (5) concomitant autoimmune diseases, psychiatric disorders, diabetes, or thyroid diseases; (6) hepatocellular carcinoma or other malignancies; (7) history of organ transplantation; (8) use of immunosuppressive agents; (9) pregnancy or planned pregnancy within two years; (10) alcohol or drug abuse; (11) co-infection with HIV, HCV, HAV, HEV, or other liver diseases; or (12) any other condition deemed unsuitable for interferon therapy.

### 2.2. Data Collection and Definitions

Data collection included case ID, sex, age, diagnosis, testing time, HBsAg levels, HBeAg status, alanine aminotransferase (ALT) levels, baseline neutrophil counts, and HBV DNA load. HBsAg and HBeAg were quantified using the ARCHITECT i2000SR and AxSYM systems (Abbott, IL, USA). Serum alanine aminotransferase (ALT) levels and other biochemical parameters were measured using a VITROS 5600 Integrated System (Ortho-Clinical Diagnostics, Rochester, NY, USA). Peripheral blood cell counts, including neutrophil counts, were determined using a BC-6900 automated hematology analyzer (Mindray, Shenzhen, China). Serum HBV DNA was quantified using fluorescence-based quantitative polymerase chain reaction (qPCR). Nucleic acids were extracted using a magnetic bead-based method, and HBV DNA extraction and amplification/detection were performed using reagents supplied by Livzon (Zhuhai, China), according to the manufacturer’s instructions.

### 2.3. Outcome Measures

We collected data from 24 weeks prior to treatment initiation and continued follow-up for up to 48 weeks after treatment, with HBsAg loss events recorded during the observation period. The primary outcome was the longitudinal trajectory of serum HBsAg levels in patients with CHB, and the secondary outcome was HBsAg loss during follow-up. HBsAg loss was used primarily as a dichotomous grouping variable to characterize differences in longitudinal HBsAg trajectories between patients with and without loss, rather than to estimate the population-level efficacy of IFN-based therapy. Consequently, the observed proportion is a descriptive summary of this particular cohort and should not be generalized as a treatment-effect estimate.

HBeAg negativity was defined as <1 S/CO; otherwise, patients were considered HBeAg-positive. HBV DNA ≥ 100 IU/mL (lower limit of detection) was defined as viremia, whereas values below this threshold were defined as non-viremia. Among viremic patients, male alanine aminotransferase (ALT) > 30 IU/L or female ALT > 19 IU/L was defined as the immune-active phase; otherwise, patients were classified as immune-inactive. HBsAg loss was defined as sustained HBsAg levels < 0.05 IU/mL with concomitant HBeAg and HBV DNA negativity. Persistent HBsAg ≥ 0.05 IU/mL was defined as sustained positivity, while all other patterns were classified as serological fluctuation or reactivation [3,4].

### 2.4. Statistical Analysis

Statistical analyses were performed using Excel 2021, MATLAB R2018a, IBM SPSS version 22, and Python version 3.9.12. Differences in weekly treatment efficacy between groups were assessed using the Kruskal–Wallis test, while trends in HBsAg decline were analyzed using generalized estimating equations (GEEs).

Quantitative HBsAg levels were log10-transformed for longitudinal analyses because their values spanned several orders of magnitude, thereby facilitating visualization and comparison of HBsAg kinetics over time.

Given the non-randomized allocation of treatment regimens, propensity score matching (PSM) and stratified analyses were applied to reduce baseline imbalances and potential selection bias caused by measured confounding factors [19]. All tests were two-sided, and a *p*-value < 0.05 was considered statistically significant.

## 3. Results

### 3.1. Baseline Demographics and Clinical Characteristics

A total of 533 patients with CHB were enrolled in this study and categorized into two groups based on HBsAg loss status (Table 1): the non-HBsAg loss group (*n* = 460) and the HBsAg loss group (*n* = 73). Baseline demographic and clinical characteristics were well balanced between the two groups, with no significant differences observed in sex, age, family history of hepatocellular carcinoma, liver cirrhosis, body mass index, neutrophil count, or treatment-naïve status (all *p* > 0.05).

Regarding liver inflammatory status, the non-HBsAg loss group exhibited a higher proportion of elevated alanine aminotransferase (ALT) levels and active hepatitis (*p* = 0.006 and *p* < 0.001, respectively). In addition, treatment regimens differed significantly between the two groups (*p* = 0.024): interferon (IFN) monotherapy was more frequently administered in the HBsAg loss group, whereas IFN combined with tenofovir alafenamide (TAF) or entecavir (ETV) was predominant in the non-HBsAg loss group. Detailed baseline comparisons are presented in Table 1.

**Figure 1:**
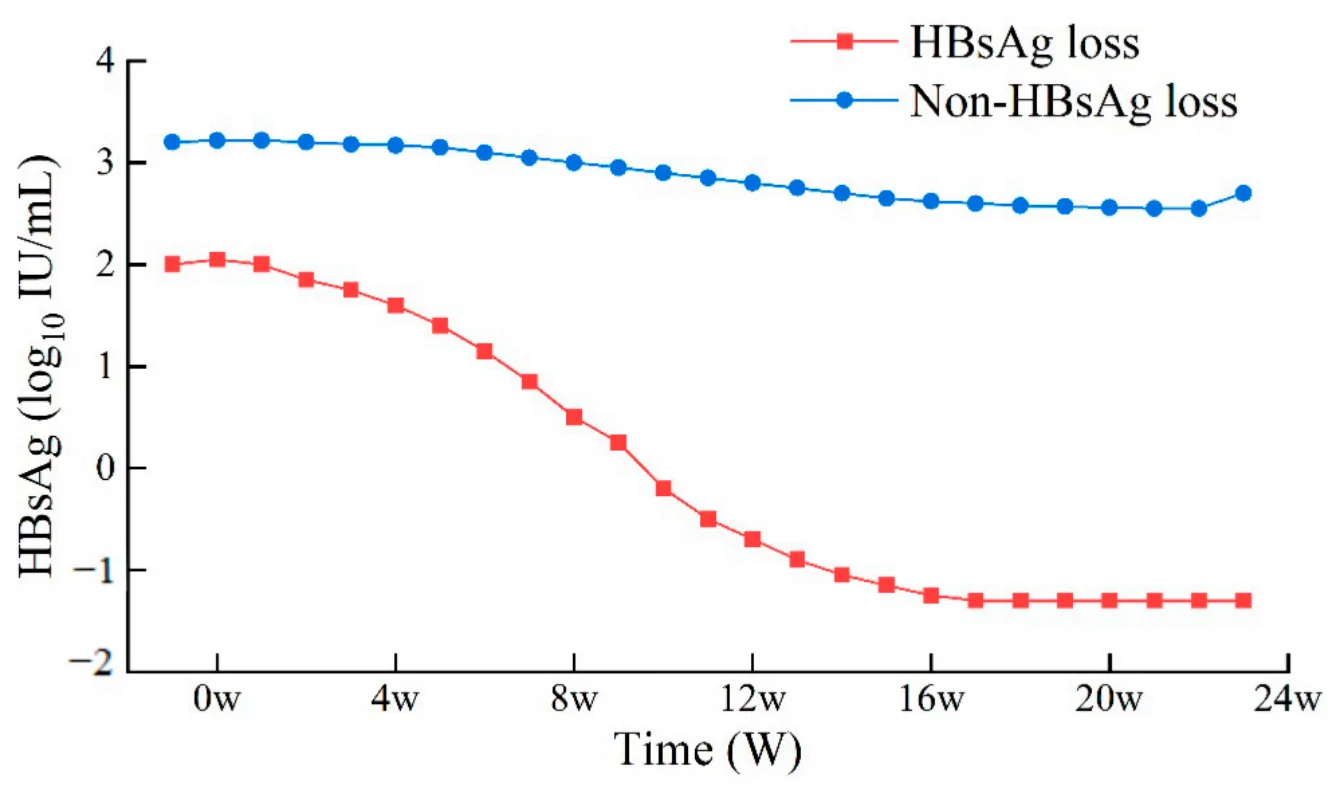
HBsAg kinetics stratified by treatment outcome. HBsAg levels are presented as log10 IU/mL over the 24-week observation period in patients with and without HBsAg loss. Patients who achieved HBsAg loss exhibited a more rapid decline than those who did not. Negative values on the log10 scale represent HBsAg concentrations below 1 IU/mL. Longitudinal differences between groups were evaluated using GEE analysis.

As shown in Figure 1, patients who achieved HBsAg loss (73 cases, 13.7%) had a relatively lower baseline HBsAg level (~10^2^ IU/mL). In this cohort, HBsAg levels declined rapidly in this group, with a marked reduction observed by week 12 and the group-level HBsAg trajectory reaching the HBsAg loss threshold by approximately week 16. As described in the Materials and Methods Section, we acknowledge that retaining patients with early HBsAg clearance enriches the cohort for responders, thereby inflating the crude clearance proportion. However, because our primary analyses focus on trajectory patterns conditional on the final outcome status and incorporate propensity score matching to balance baseline characteristics, this selection does not affect the comparative trajectory estimates. Nonetheless, the absolute clearance rate reported here should be interpreted with caution and only within the context of this selected cohort.

In contrast, patients without HBsAg loss (460 cases, 86.3%) had a higher baseline HBsAg level (~10^3.2^ IU/mL) and exhibited a slow and approximately linear decline over 24 weeks, remaining at approximately 10^2.7^ IU/mL at week 24. A slight fluctuation was observed toward the end of the 24-week observation period; however, this did not alter the overall declining HBsAg trajectory.

**Figure 2:**
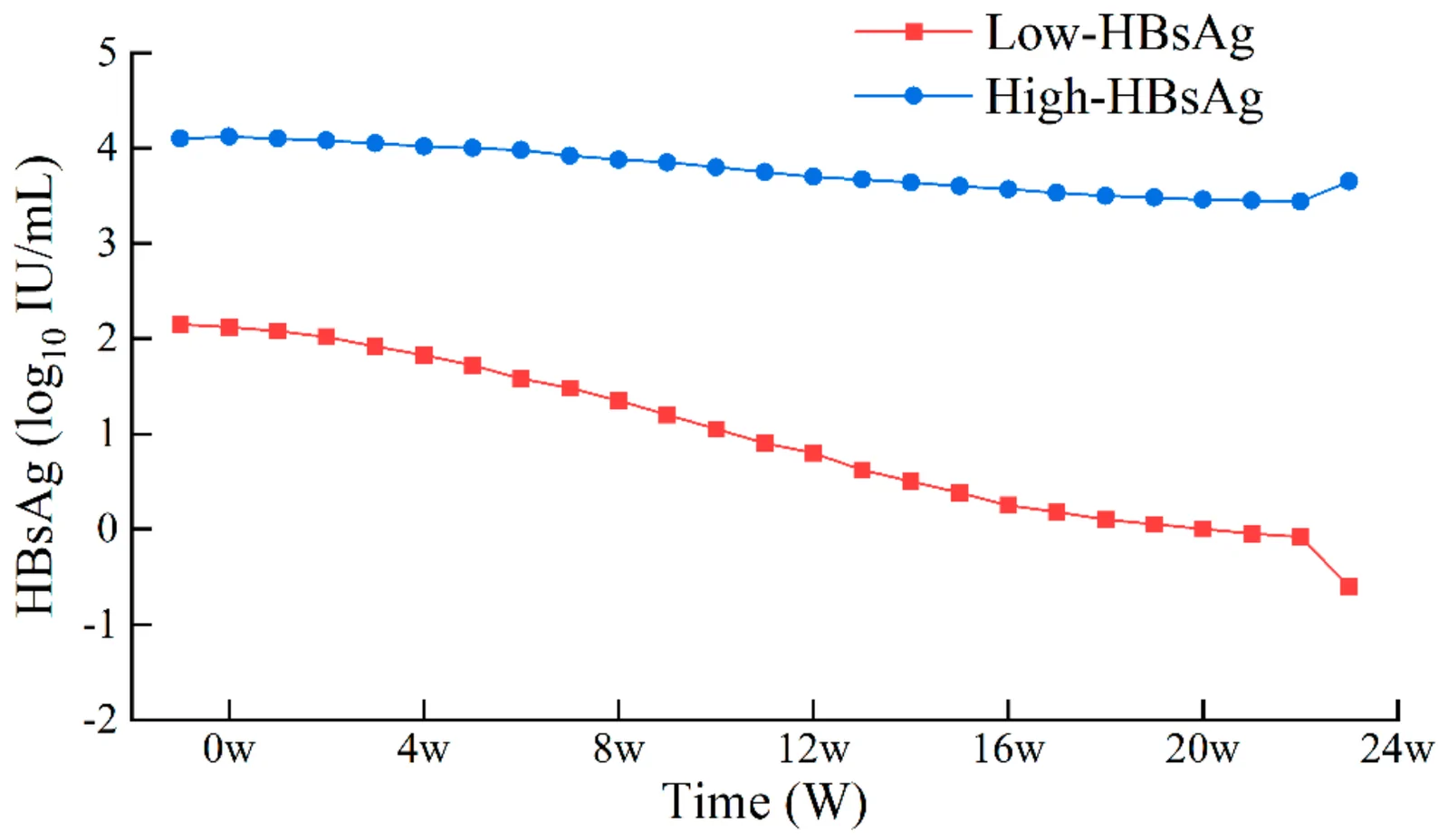
HBsAg kinetics stratified by baseline HBsAg level. Patients were stratified according to the median baseline HBsAg level (3.11 log10 IU/mL). The low-baseline-HBsAg group showed a greater overall decline, whereas the high-baseline-HBsAg group exhibited a more modest reduction. A slight late fluctuation was observed toward week 24, particularly in the high-baseline-HBsAg group, while the overall trajectory remained below baseline. Longitudinal differences were evaluated using GEE analysis.

**Figure 3:**
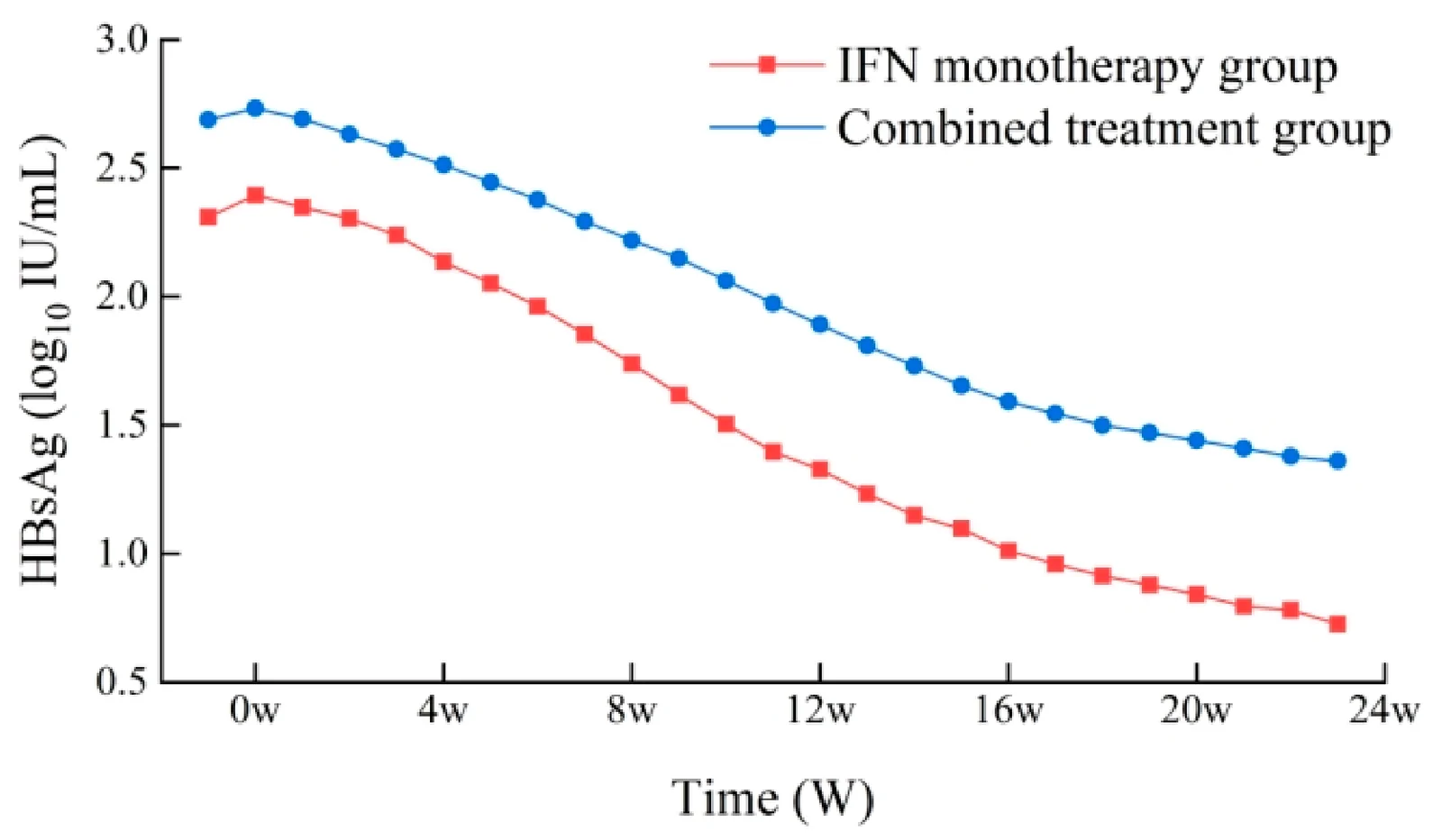
HBsAg kinetics stratified by treatment strategy (after PSM). HBsAg levels are presented as log10 IU/mL over the 24-week observation period in patients receiving IFN monotherapy or IFN plus nucleos(t)ide analog combination therapy. The two groups showed comparable overall longitudinal decline patterns. Longitudinal differences were evaluated using generalized estimating equation (GEE) analysis.

**Figure 4:**
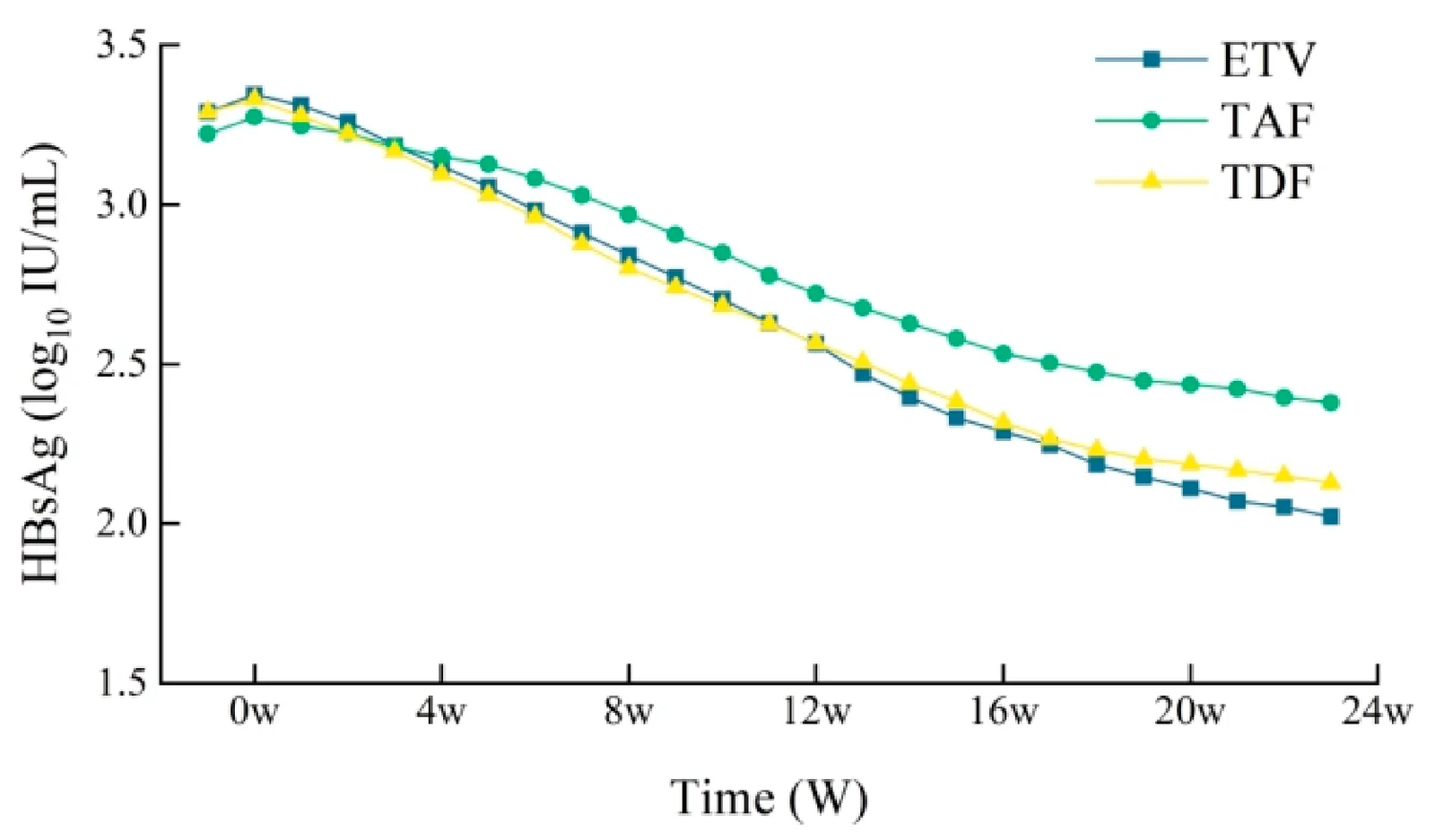
HBsAg kinetics stratified by combination therapy regimens (after PSM): comparison among IFN + ETV, IFN + TAF, and IFN + TDF groups. The three regimens showed broadly similar overall HBsAg decline trajectories. Longitudinal differences were evaluated using GEE analysis.

An apparent transient increase in HBsAg was also observed during the early treatment period, followed by a subsequent decline. Similar early patterns were observed across different treatment and clinical strata in the subsequent analyses (Figure 1, Figure 2, Figure 3 and Figure 4). Given the descriptive and exploratory nature of this observation, it was interpreted cautiously.

GEE analysis demonstrated that patients who achieved HBsAg loss exhibited a significantly faster decline in HBsAg levels over time compared with those who did not achieve HBsAg loss (*p* < 0.001). The longitudinal trajectories of HBsAg differed significantly between the two groups across all time points (*p* < 0.001). Overall, Figure 1 demonstrates distinct longitudinal patterns, characterized by a rapid decline toward HBsAg loss in patients who subsequently achieved HBsAg loss and a slower decline with persistent HBsAg positivity in those who did not.

### 3.2. Impact of Baseline HBsAg Levels on HBsAg Kinetics and HBsAg Loss

Previous studies have established that baseline HBsAg levels are a critical determinant of therapeutic efficacy. Accordingly, patients were stratified according to baseline HBsAg levels. In the low-HBsAg group (below the median, <3.11 log_10_ IU/mL, approximately 1288 IU/mL; n = 267), HBsAg levels declined from approximately 10^2.1^ IU/mL at baseline to 10^0.8^ IU/mL at week 24, corresponding to an average reduction of approximately 1.33 log_10_ IU/mL. Separately, 22.8% of patients in this low-baseline-HBsAg group achieved HBsAg loss during follow-up. In contrast, the high-HBsAg group (≥median; n = 266) showed only a modest decline, from approximately 10^4.0^ IU/mL at baseline to 10^3.6^ IU/mL at week 24, representing a reduction of 0.49 log_10_ IU/mL. HBsAg loss was observed in 4.5% of patients in this group (*p* < 0.001).

As shown in Figure 2, the low-baseline-HBsAg group exhibited a greater and more sustained overall decline in HBsAg during the 24-week observation period, whereas the high-baseline-HBsAg group showed a smaller overall reduction and persistently higher HBsAg levels. A slight fluctuation was also observed toward week 24, particularly in the high-baseline-HBsAg group; however, HBsAg levels remained below baseline, and the overall 24-week trajectory was still characterized by a net decline. GEE analysis demonstrated that the rate of HBsAg decline over time was significantly more rapid in the low-baseline-HBsAg group compared to the high-baseline-HBsAg group.

These findings indicate that baseline HBsAg is a crucial determinant of both dynamic kinetics and clinical cure outcomes, with distinct 24-week trajectories characterizing patients who eventually achieve HBsAg loss versus those who do not.

### 3.3. Comparison of HBsAg Kinetics Across Different Treatment Strategies After Matching

Before PSM, 97 patients received IFN monotherapy, and 436 received IFN plus NA combination therapy. Significant differences in several baseline characteristics were observed between the two groups. Compared with the combination therapy group, the monotherapy group had lower baseline HBsAg levels, a higher proportion of treatment-naïve patients (67.0% vs. 44.7%), a lower proportion of HBeAg-positive patients (32.0% vs. 49.5%), and a lower prevalence of cirrhosis (5.2% vs. 13.5%) (Table 2). These baseline differences indicated potential confounding in the unadjusted comparison and therefore supported the use of PSM to improve comparability between the two treatment groups.

Therefore, PSM was performed to compare HBsAg kinetics over 24 weeks between patients receiving IFN monotherapy and those receiving combination therapy. Following PSM, 84 patients in the monotherapy group and 177 patients in the combination therapy group were retained. Baseline characteristics were well balanced between the two groups, with no statistically significant differences observed (Table 2). It is also important to recognize that PSM, while effective in reducing measured confounding, substantially reduced the sample size and altered the composition of the analyzed cohort. The matched population represents a subset of patients with comparable baseline characteristics across treatment groups, which enhances internal validity for trajectory comparisons but may limit external generalizability—particularly to patients at the extremes of the propensity distribution. The reduced statistical power may have precluded detection of modest but clinically relevant differences. These trade-offs should be kept in mind when interpreting the comparative findings from the matched analyses.

The median weekly HBsAg trajectories of the matched cohorts are illustrated in Figure 3. Both groups exhibited a similar overall declining pattern, characterized by relative stability or mild fluctuation during the early treatment phase (weeks 2–4), followed by a sustained decline thereafter. Notably, HBsAg levels in the IFN monotherapy group remained consistently lower than those in the combination therapy group throughout follow-up, ranging from approximately 2.3 log_10_ IU/mL versus 2.7 log_10_ IU/mL at baseline to approximately 0.7 log_10_ IU/mL versus 1.4 log_10_ IU/mL at week 24. This difference persisted across the entire observation period.

GEE analysis of the matched cohort demonstrated that, after adjusting for baseline HBsAg levels, time had a significant effect on HBsAg decline in both groups (*p* < 0.001). However, no statistically significant main effect of the treatment regimen was observed (*p* = 0.350). Furthermore, the interaction between treatment regimen and time was not significant (*p* = 0.100), indicating that the longitudinal HBsAg decline patterns were comparable between the two groups over the 24-week follow-up period. These results indicate that HBsAg levels declined significantly over time in both groups, whereas the overall longitudinal HBsAg trajectories did not differ significantly between IFN monotherapy and IFN plus NA combination therapy after matching.

### 3.4. Comparison of HBsAg Kinetics Among Three Combination Therapy Regimens After Matching

To further investigate dynamic changes in HBsAg levels during treatment among the three combination regimens (IFN + ETV, IFN + TAF, and IFN + TDF), patients receiving combination therapy were included in subsequent analyses. Baseline characteristics revealed significant imbalances among the three groups across multiple variables, including sex distribution, age, proportion of elevated ALT, HBeAg positivity rate, HBV DNA viremia status, treatment-naïve status, and prevalence of active hepatitis (all *p* < 0.05). In contrast, no statistically significant differences were observed in cirrhosis, baseline HBsAg levels, body mass index, or neutrophil count (all *p* > 0.05).

To mitigate confounding bias and enhance comparability among the groups, a 1:1:1 PSM approach was applied. Before matching, the sample sizes were 198 in the IFN + TDF group, 96 in the IFN + TAF group, and 142 in the IFN + ETV group. After 1:1:1 PSM, 73 patients were retained in each group. PSM improved the overall comparability of baseline characteristics among the three treatment groups; however, sex remained significantly imbalanced after matching (*p* = 0.007). Given the potential influence of sex on HBsAg dynamics, this residual imbalance should be considered when interpreting the comparisons among the three treatment regimens (Table 3).

Post-matching baseline covariates demonstrated substantial improvement, as evidenced by reduced standardized mean differences (SMDs) across most variables, indicating enhanced overall balance among the three groups. No statistically significant differences were observed in the majority of clinical and laboratory variables after matching, supporting acceptable baseline comparability for subsequent analyses. PSM improved comparability across the three treatment groups, although the reduced sample size should be considered when interpreting the post-matching comparisons.

Notably, the cumulative HBsAg loss rates among the three treatment regimens were not significantly different before and after PSM, suggesting comparable efficacy of the three combination therapies in achieving HBsAg loss.

The median weekly HBsAg trajectories of the three matched groups are illustrated in Figure 4, demonstrating an overall consistent downward trend across all regimens. During the early treatment phase (weeks 0–4), HBsAg levels were comparable among the three groups, followed by a sustained decline thereafter.

Descriptively, a more pronounced decline was observed in the IFN + ETV and IFN + TDF groups, with median HBsAg levels decreasing to approximately 2.0 log_10_ IU/mL and 2.1 log_10_ IU/mL, respectively, by week 24. In contrast, the IFN + TAF group showed a relatively slower decline, with median HBsAg levels remaining at approximately 2.4 log_10_ IU/mL at week 24.

However, GEE analysis demonstrated no statistically significant interaction between treatment regimen and time among the three groups, suggesting that the longitudinal trajectories of HBsAg decline were comparable across regimens over the 24-week follow-up period.

### 3.5. Stratified Analysis Based on Clinical Characteristics, HBV DNA and HBeAg Status

Based on the 24-week changes in HBsAg levels, the therapeutic effects of the three combination regimens (IFN + TDF, IFN + TAF, and IFN + ETV) were further evaluated in subgroups stratified by sex, disease phase, HBV DNA status (detectable/undetectable), HBeAg status (positive/negative), and treatment history. GEE models were used to assess whether the interaction between treatment regimen and time differed among subgroups.

**Figure 5:**
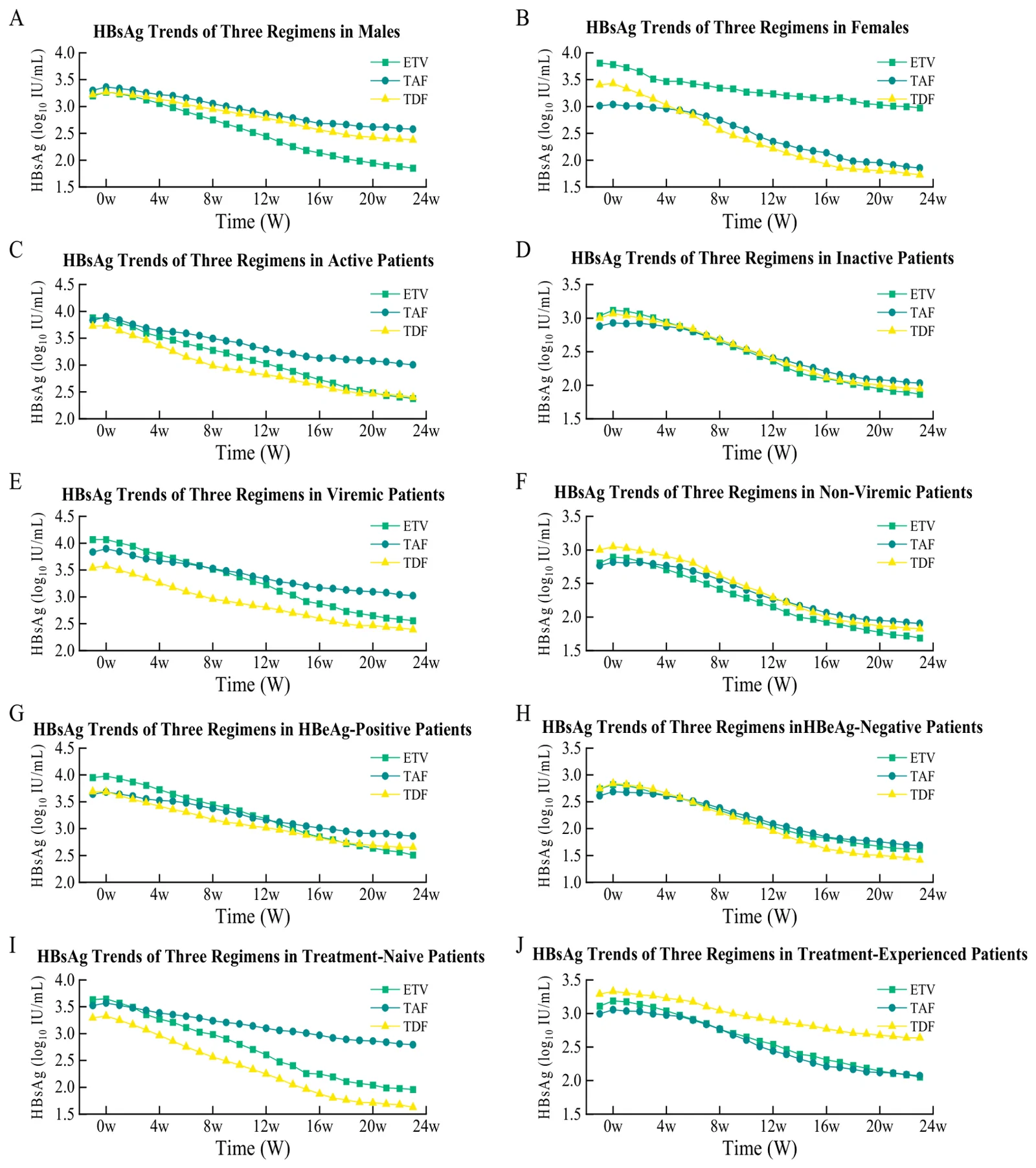
HBsAg kinetics of three antiviral regimens across patient subgroups after PSM. HBsAg levels are presented as log10 IU/mL. Panels show analyses stratified by sex, immune activity, HBV DNA status, HBeAg status, and treatment history. Panel-specific y-axis ranges were retained to improve visualization of within-subgroup longitudinal changes. Treatment-by-time interactions were assessed using GEE models. (**A**) Male patients; (**B**) female patients; (**C**) active hepatitis; (**D**) inactive hepatitis; (**E**) viremic patients; (**F**) non-viremic patients; (**G**) HBeAg-positive; (**H**) HBeAg-negative; (**I**) treatment-naïve; (**J**) treatment-experienced.

In male patients (Figure 5A), the HBsAg decline trajectories among the three regimens were generally comparable. However, the ETV group demonstrated a significantly faster rate of HBsAg decline compared with the TAF group, with the divergence becoming more apparent after week 18 and persisting through week 24.

In female patients (Figure 5B), although no significant differences in absolute HBsAg levels were observed between the TDF and ETV groups within 24 weeks, GEE analysis indicated a significantly faster rate of HBsAg decline in the TDF group compared with the ETV group.

Among patients in the immune-active phase (Figure 5C), the ETV group exhibited a relatively faster rate of HBsAg decline compared with the TAF group, with this difference mainly emerging between weeks 21 and 24. In contrast, patients in the immune-inactive phase (Figure 5D) showed no statistically significant differences in HBsAg decline trajectories among the three regimens.

For patients with detectable HBV DNA levels (viremia, Figure 5E), the ETV group showed a relatively faster rate of HBsAg decline compared with the TAF group; however, no statistically significant differences in absolute HBsAg levels were observed between these two groups during the 24-week follow-up period. Similarly, among patients without viremia (Figure 5F), no significant differences in HBsAg decline trajectories were observed among the three regimens.

Regarding HBeAg status, comparable HBsAg decline patterns were observed among HBeAg-positive (Figure 5G) and HBeAg-negative patients (Figure 5H), with no statistically significant differences among the three treatment regimens.

Finally, in treatment-naïve patients (Figure 5I), the three regimens showed different numerical patterns of HBsAg decline; however, no significant treatment-by-time interaction was observed among the groups. In treatment-experienced patients (Figure 5J), the ETV group exhibited a faster rate of HBsAg decline compared with the TAF group, while TDF also demonstrated a faster decline rate than TAF; however, no significant differences in absolute HBsAg levels were observed between the corresponding groups within 24 weeks.

**Figure 6:**
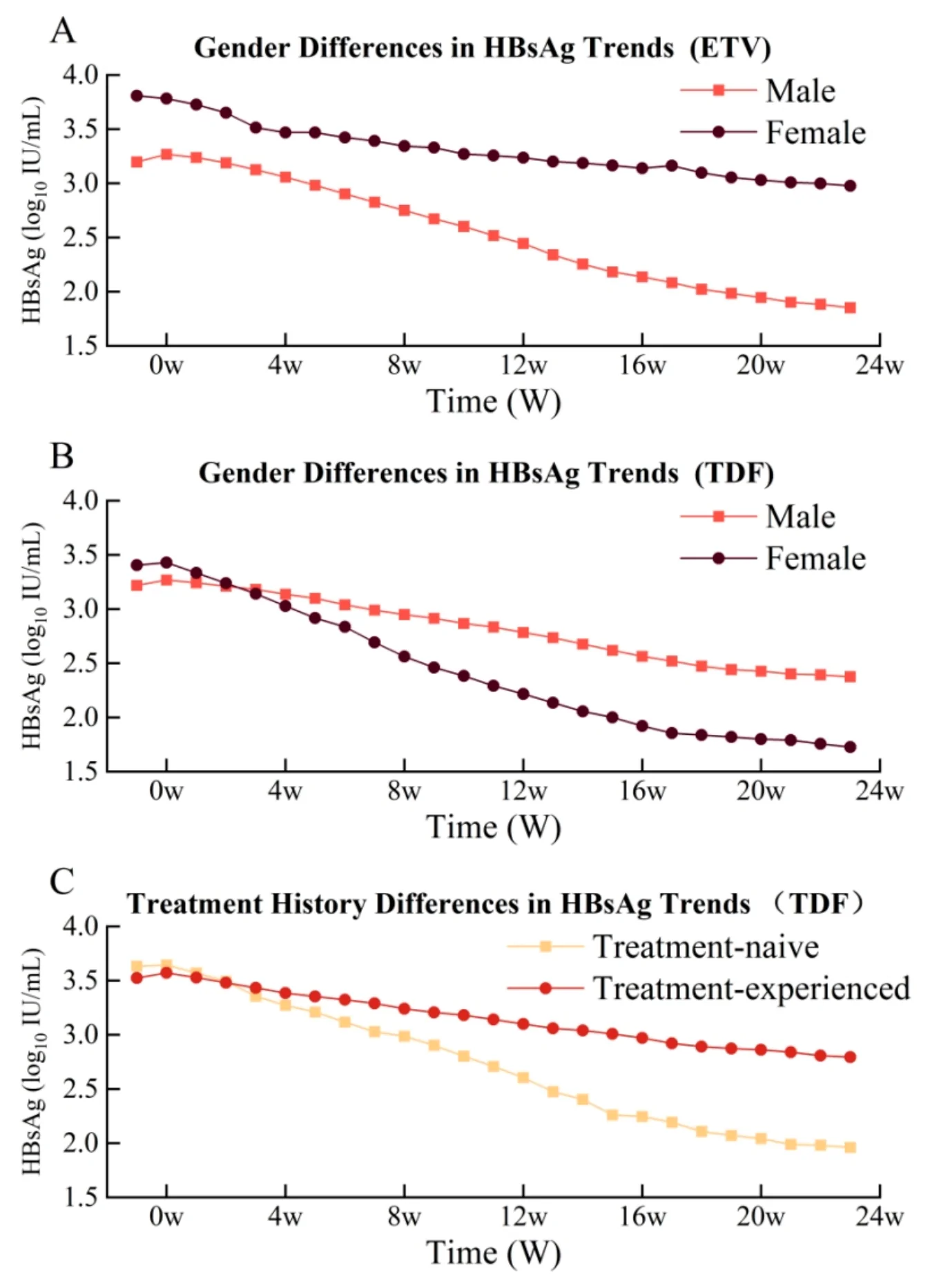
Subgroup analysis of HBsAg kinetics by gender and treatment history. HBsAg levels are presented as log10 IU/mL over the 24-week observation period. Panel A compares male and female patients receiving IFN + ETV; Panel B compares male and female patients receiving IFN + TDF; and Panel C compares treatment-naïve and treatment-experienced patients receiving IFN + TDF. Longitudinal differences were evaluated using GEE analysis. (**A**) Gender differences in the ETV group; (**B**) gender differences in the TDF group; (**C**) treatment history differences in the TDF group.

Further subgroup analyses examining the effects of individual drugs across different patient populations revealed distinct patterns of HBsAg decline. ETV was associated with a faster rate of HBsAg decline in male patients compared with female patients (Figure 6A), with this difference becoming more apparent from week 18 onward. In contrast, TDF demonstrated a faster rate of HBsAg decline in female patients compared with male patients (Figure 6B). However, no statistically significant differences in absolute HBsAg levels were observed between sexes within the 24-week follow-up period. In addition, TDF appeared to exhibit a faster rate of HBsAg decline in treatment-experienced patients compared with treatment-naïve patients (Figure 6C); however, no statistically significant differences in HBsAg levels were observed between the two groups during the 24-week observation period.

## 4. Discussion

### 4.1. HBsAg Kinetics Heterogeneity and Prognostic Value

The dynamic changes in HBsAg during treatment reflect the complex interplay among intrahepatic viral replication and transcriptional activity, host immune responses, and hepatocyte status in CHB. HBsAg kinetics have been widely recognized as a key biomarker for predicting long-term treatment outcomes, particularly functional cure. Recent studies and meta-analyses have further confirmed that early on-treatment HBsAg decline is strongly associated with subsequent HBsAg loss, supporting the use of longitudinal HBsAg monitoring for individualized treatment assessment [9]. For example, Itokawa N et al. [20] reported that baseline HBsAg levels, together with early on-treatment decline, jointly determine therapeutic outcomes.

In the present study, we observed marked heterogeneity in HBsAg kinetics across patients with different outcomes. Patients who achieved HBsAg loss exhibited a characteristic accelerated decline pattern, whereas those without HBsAg loss showed a slow and nearly linear decline trajectory. These distinct kinetic phenotypes persisted throughout the entire follow-up period, supporting the clinical relevance of HBsAg dynamic stratification in CHB.

Further analyses demonstrated that the baseline HBsAg level is a key determinant of both kinetic trajectory and final treatment outcome. Patients with lower baseline HBsAg levels exhibited more rapid decline and significantly higher HBsAg loss rates within 24 weeks, whereas those with higher baseline levels showed only modest reductions and lower clearance rates. These findings are consistent with previous reports. Marcellin P et al. [21] demonstrated that early rapid HBsAg decline is strongly associated with subsequent HBsAg loss in HBeAg-positive patients. Similarly, in HBeAg-negative populations, low-baseline-HbsAg levels (particularly < 100 IU/mL) have been linked with more favorable antigen reduction profiles [22]. Li MH et al. [23] further revealed that patients achieving HBsAg clearance exhibit pronounced early declines during treatment, reinforcing the predictive value of early HBsAg kinetics.

### 4.2. IFN Monotherapy vs. Combination Therapy: Impact of Selection Bias

In the unadjusted real-world cohort, patients receiving IFN monotherapy exhibited a higher HBsAg loss rate than those receiving combination therapy (22.7% vs. 7.1%). At first glance, this finding appears to suggest the superior efficacy of monotherapy. However, further analyses indicate that this difference is largely attributable to baseline imbalances rather than treatment strategies.

Specifically, patients in the IFN monotherapy group had more favorable baseline characteristics, including lower HBsAg levels, a higher proportion of treatment-naïve individuals, a greater proportion of HBeAg-negative patients, and a lower prevalence of cirrhosis. These factors are well-established predictors of HBsAg decline and functional cure. In routine clinical practice, such patients are more likely to be selected for IFN monotherapy, whereas combination therapy is typically reserved for individuals with more advanced or less favorable disease profiles, leading to confounding by indication.

After PSM, the rate of HBsAg decline was comparable between treatment strategies, and no significant difference in HBsAg loss rates was observed. Mechanistically, HBsAg clearance is primarily driven by interferon-mediated immune modulation, whereas NAs mainly suppress viral replication. Recent studies have suggested that adding Peg-IFN to NA therapy may enhance HBsAg decline and increase the probability of a functional cure in selected patients; however, the benefit remains heterogeneous [5]. Consistent with this, a meta-analysis of 49 clinical trials demonstrated similar efficacy between IFN monotherapy and combination therapy in terms of ALT normalization, HBeAg loss, and HBsAg clearance, although combination therapy showed superior HBV DNA suppression [24].

### 4.3. Heterogeneity Among NA-Based Combination Regimens

Subgroup analyses showed that IFN-based combination regimens exhibited broadly similar HBsAg decline patterns in most clinical settings, suggesting that the choice of NAs is not the primary determinant of treatment response. No significant differences were observed among regimens when patients were stratified by immune status, viral load, treatment history, or HBeAg status.

However, heterogeneity was observed in specific subgroups. In female patients, TDF was associated with a faster rate of HBsAg decline compared with ETV, whereas in male patients, ETV showed faster kinetics than TAF. Similarly, in immune-active and viremic patients, ETV exhibited a more rapid decline than TAF, while in treatment-experienced patients, TDF demonstrated a faster decline than TAF. Importantly, these differences were observed in kinetic slopes rather than absolute HBsAg levels over the 24-week period, suggesting early divergence in viral dynamics rather than definitive differences in clinical efficacy.

These findings support the concept that HBsAg kinetics under IFN-based combination therapy are shaped by context-dependent interactions between antiviral agents and host factors. In conditions characterized by enhanced immune activity or dynamic viral reservoirs, such as immune-active disease or prior treatment exposure, subtle pharmacologic differences among NAs may be amplified, leading to measurable differences in decline rates.

In addition, differential host responsiveness may further contribute to the observed heterogeneity. Previous studies have shown that females exhibit stronger type I interferon responses [25], whereas males may demonstrate more favorable responses to NA-based therapy [26]. The present study provides real-world evidence of how such biological differences may manifest under combination therapy, highlighting the importance of host–drug interactions in CHB management.

### 4.4. Early Transient Increase in HBsAg

An apparent transient increase in HBsAg was observed during the early phase of IFN-based therapy, characterized by an initial increase above baseline followed by a subsequent decline. Notably, similar early patterns were observed across subgroups defined by different treatment regimens and clinical characteristics. Therefore, this finding should be interpreted with caution.

The relatively consistent pattern across different subgroups suggests that non-biological factors cannot be excluded as potential contributors to this observation. Possible explanations include assay- or dilution-related effects, batch variation, sample-processing procedures, and the handling of longitudinal measurements. Because the HBsAg measurements in this study were retrospectively obtained from routine clinical practice, these potential technical or pre-analytical factors could not be fully evaluated or excluded. Therefore, the observed early increase in HBsAg should not be directly interpreted as a specific biological response to IFN or nucleos(t)ide analog therapy.

Nevertheless, potential biological mechanisms remain plausible and warrant further investigation. IFN-induced activation of innate and adaptive immune responses, including changes in natural killer (NK) cell and CD8+ T-cell activity, may influence hepatocyte turnover and circulating viral antigen levels [27]. In addition, experimental studies have suggested that antiviral therapy may affect HBV transcriptional activity or cccDNA-related regulation [28,29]. These previous findings provide biological plausibility for short-term fluctuations in circulating HBsAg; however, they do not establish the mechanism underlying the early HBsAg increase observed in our cohort. Thus, the early increase in HBsAg observed in this study should be regarded as an exploratory finding, and therefore no causal inference regarding its underlying mechanism can be made. Further prospective studies are needed to clarify its potential mechanism.

## 5. Conclusions

Based on intensive longitudinal monitoring, this study revealed substantial heterogeneity in HBsAg kinetics among patients with CHB and identified an apparent transient increase in HBsAg during the early phase of IFN-based therapy. During the 24-week treatment period, an early rapid decline in HBsAg was associated with a higher likelihood of achieving HBsAg loss, and patients with lower baseline HBsAg levels were more likely to experience a faster reduction. After PSM, IFN monotherapy and combination therapy exhibited similar HBsAg decline trajectories, and comparable patterns were also observed among the three combination regimens. Notably, subgroup analyses revealed significant heterogeneity in HBsAg kinetics within combination therapies. Treatment responses to IFN and different NAs varied according to sex and disease stage, indicating a context-dependent pattern of antiviral response.

Overall, these findings highlight the importance of precise and dynamic monitoring of HBsAg levels, providing valuable insights for the accurate assessment of treatment response, optimization of individualized therapeutic strategies, and development of more robust prognostic models for CHB.

## Figures and Tables

**Table 1:** Baseline characteristics of enrolled patients.

Variables	Non-HBsAg Loss (*n* = 460)	HBsAg Loss (*n* = 73)	*p*
Male sex, *n* (%)	346 (75.2%)	53 (72.6%)	0.632
Age, years, median (IQR)	37.00 (32.00, 44.00)	35.00 (31.00, 43.00)	0.116
Family history of HCC, *n* (%)	22 (4.8%)	1 (1.4%)	0.183
Cirrhosis, *n* (%)	55 (12.0%)	9 (12.3%)	0.928
HBsAg, log_10_ IU/mL, median (IQR)	3.22 (2.67, 3.82)	1.92 (1.11, 2.68)	<0.001
Elevated ALT, *n* (%)	292 (63.5%)	34 (46.6%)	0.006
HBeAg positivity, *n* (%)	226 (49.1%)	22 (30.1%)	0.003
BMI, kg/m^2^, median (IQR)	23.10 (20.90, 25.30)	23.00 (20.20, 25.60)	0.688
Neutrophil count, ×10^9^/L, median (IQR)	3.10 (2.40, 3.80)	3.20 (2.60, 3.90)	0.162
HBV DNA viremia, *n* (%)	214 (46.5%)	15 (20.5%)	<0.001
Treatment-naïve, *n* (%)	223 (48.5%)	37 (50.7%)	0.726
Active hepatitis, *n* (%)	182 (39.6%)	11 (15.1%)	<0.001
Treatment regimen, *n* (%)			0.024
IFN monotherapy	75 (16.3%)	22 (30.1%)	
IFN + TDF	172 (37.4%)	26 (35.6%)	
IFN + TAF	89 (19.3%)	7 (9.6%)	
IFN + ETV	124 (27.0%)	18 (24.7%)	

**Table 2:** Baseline characteristics by treatment strategy before and after PSM.: IFN monotherapy versus combination therapy.

Variables	Before Matching				After Matching			
	Monotherapy (*n* = 97)	Combination (*n* = 436)	*p*	SMD	Monotherapy (*n* = 84)	Combination (*n* = 177)	*p*	SMD
Male sex, *n* (%)	73 (75.3%)	326 (74.8%)	0.92	0.011	66 (78.6%)	141 (79.7%)	0.839	−0.023
Age, years, median (IQR)	35.00 (31.00, 42.00)	37.00 (32.00, 45.00)	0.83	−0.219	36.00 (31.00, 42.00)	37.00 (32.00, 45.00)	0.572	0.03
Family history of HCC, *n* (%)	1 (1.0%)	22 (5.0%)	0.078	−0.395	1 (1.2%)	2 (1.1%)	0.966	−0.039
Cirrhosis, *n* (%)	5 (5.2%)	59 (13.5%)	0.022	−0.377	5 (6.0%)	12 (6.8%)	0.8	0.018
HBsAg, log10 IU/mL, median (IQR)	2.35 (1.50, 3.03)	3.24 (2.66, 3.89)	<0.001	−0.879	2.53 (1.58, 3.11)	2.88 (1.99, 3.49)	0.258	−0.086
Elevated ALT, *n* (%)	43 (44.3%)	164 (37.6%)	0.22	−0.134	49 (58.3%)	106 (59.9%)	0.811	<0.001
HBeAg positivity, *n* (%)	31 (32.0%)	216 (49.5%)	<0.001	−0.38	28 (33.3%)	70 (39.5%)	0.333	−0.004
BMI, kg/m^2^, median (IQR)	23.10 (20.90, 26.10)	23.00 (20.80, 25.23)	0.313	0.158	23.00 (20.90, 26.12)	23.50 (21.30, 25.50)	0.989	0.033
Neutrophil count, ×10^9^/L, median (IQR)	3.20 (2.70, 4.10)	3.10 (2.40, 3.80)	0.107	0.133	3.10 (2.68, 4.10)	3.20 (2.50, 3.90)	0.876	−0.018
HBV DNA viremia	36 (37.1%)	193 (44.3%)	0.198	−0.147	34 (40.5%)	85 (48.0%)	0.253	−0.086
Treatment-naïve, *n* (%)	65 (67.0%)	195 (44.7%)	<0.001	0.472	53 (63.1%)	109 (61.6%)	0.814	−0.101
Active hepatitis, *n* (%)	29 (29.9%)	164 (37.6%)	0.153	−0.168	27 (32.1%)	73 (41.2%)	0.158	−0.112

**Table 3:** Baseline characteristics by treatment strategy before and after PSM among the IFN + TDF, IFN + TAF, and IFN + ETV groups.

Variables	Before Matching				After Matching					
	TDF (*n* = 198)	TAF (*n* = 96)	ETV (*n* = 142)	*p*	SMD	TDF (*n* = 73)	TAF (*n* = 73)	ETV (*n* = 73)	*p*	SMD
Male sex, *n* (%)	138 (69.7%)	69 (71.9%)	119 (78.3%)	0.01	0.226	45 (61.6%)	53 (72.6%)	62 (84.9%)	0.007	0.362
Age, years, median (IQR)	36.00 (32.00, 43.00)	36.00 (31.00, 44.00)	40.00 (35.00, 47.00)	0.007	0.134	37.00 (28.00, 48.00)	34.00 (31.00, 43.00)	38.00 (31.00, 47.00)	0.572	0.102
Family history of HCC, *n* (%)	15 (7.6%)	3 (3.1%)	4 (2.6%)	0.088	0.144	3 (4.1%)	3 (4.1%)	3 (4.1%)	1	<0.001
Cirrhosis, *n* (%)	26 (13.1%)	9 (9.4%)	24 (15.8%)	0.244	0.15	11 (15.1%)	8 (11.0%)	14 (19.2%)	0.8	0.154
HBsAg, log_10_ IU/mL, median (IQR)	3.34 (2.65, 4.07)	3.31 (2.86, 3.80)	3.06 (2.64, 3.62)	0.125	0.084	3.35 (2.87, 3.82)	3.32 (2.87, 3.78)	3.19 (2.70, 4.09)	0.258	0.047
Elevated ALT, *n* (%)	145 (73.2%)	57 (59.4%)	70 (46.1%)	<0.001	0.336	47 (64.4%)	41 (56.2%)	39 (53.4%)	0.377	0.149
HBeAg positivity, *n* (%)	101 (51.0%)	56 (58.3%)	60 (39.5%)	0.046	0.217	42 (57.5%)	43 (58.9%)	33 (45.2%)	0.188	0.184
BMI, kg/m^2^, median (IQR)	23.10 (20.90, 25.38)	23.00 (20.60, 24.96)	23.45 (20.80, 25.27)	0.721	0.072	22.90 (19.70, 25.30)	22.70 (20.20, 25.50)	22.90 (20.10, 24.80)	0.989	0.011
Neutrophil count, ×10^9^/L, median (IQR)	3.10 (2.50, 3.80)	3.20 (2.27, 3.73)	2.90 (2.32, 3.68)	0.239	0.116	3.00 (2.40, 3.70)	3.20 (2.20, 3.70)	2.90 (2.20, 3.50)	0.876	0.102
HBV DNA viremia, *n* (%)	110 (55.6%)	47 (49.0%)	36 (23.7%)	<0.001	0.428	39 (53.4%)	31 (42.5%)	28 (38.4%)	0.167	0.135
Treatment-naïve, *n* (%)	122 (61.6%)	41 (42.7%)	32 (21.1%)	<0.001	0.563	37 (50.7%)	31 (42.5%)	25 (34.2%)	0.133	0.203
Active hepatitis, *n* (%)	95 (48.0%)	40 (41.7%)	29 (19.1%)	<0.001	0.402	29 (39.7%)	26 (35.6%)	22 (30.1%)	0.477	0.224
HBsAg loss, *n* (%)	26 (13.1%)	7 (7.3%)	18 (11.8%)	0.312	–	10 (13.7%)	6 (8.2%)	12 (16.4%)	0.318	–

– = Not statistically significant.

## Data Availability

The datasets generated during this study are available from the corresponding author on reasonable request.

## Abbreviations

The following abbreviations are used in this manuscript:

ALT	Alanine aminotransferase
cccDNA	Covalently closed circular DNA
CHB	Chronic hepatitis B
ETV	Entecavir
GEE	Generalized estimating equations
HBeAg	Hepatitis B e antigen
HBsAg	Hepatitis B surface antigen
HBV	Hepatitis B virus
HCC	Hepatocellular carcinoma
IFN	Interferon
NA	Nucleos(t)ide analog
Peg-IFN-α	Pegylated interferon-alpha
PSM	Propensity score matching
TAF	Tenofovir alafenamide
TDF	Tenofovir disoproxil fumarate
WHO	World Health Organization
